# A new animal product free defined medium for 2D and 3D culturing of normal and cancer cells to study cell proliferation and migration as well as dose response to chemical treatment

**DOI:** 10.1016/j.toxrep.2023.04.001

**Published:** 2023-04-12

**Authors:** Ólöf Birna Rafnsdóttir, Anna Kiuru, Mattis Tebäck, Nathalie Friberg, Philippa Revstedt, Johan Zhu, Sofia Thomasson, Agnieszka Czopek, Atena Malakpour-Permlid, Tilo Weber, Stina Oredsson

**Affiliations:** a Department of Biology, Lund University, 22362 Lund, Sweden; b Institute of Life and Environmental Sciences, School of Engineering and Natural Sciences, University of Iceland, 101 Reykjavík, Iceland; c Occupational and Environmental Dermatology, Skåne University Hospital, 214 28 Malmö, Sweden; d Clinical Microbiology and Infection Prevention and Control, Region Skåne, 221 85 Lund, Sweden; e Atos Medical AB, 242 35 Hörby, Sweden; f Center for Intelligent Drug Delivery and Sensing Using Microcontainers and Nanomechanics, Department of Health Technology, Technical University of Denmark, 2800 Kongens Lyngby, Denmark; g Animal Welfare Academy of the German Animal Welfare Federation, 85579 Neubiberg, Germany

**Keywords:** Animal product free defined medium, Cancer cells, Normal cells, Routine culturing, Growth curves, Dose response curves, Cell migration, Time-lapse imaging, Two dimensions, Three dimensions

## Abstract

Cell culturing methods are increasingly used to reduce and replace the use of live animals in biomedical research and chemical toxicity testing. Although live animals are avoided when using cell culturing methods, they often contain animal-derived components of which one of the most commonly used is foetal bovine serum (FBS). FBS is added to cell culture media among other supplements to support cell attachment/spreading and cell proliferation. The safety, batch-to-batch variation, and ethical problems with FBS are acknowledged and therefore world-wide efforts are ongoing to produce FBS free media. Here, we present the composition of a new defined medium with only human proteins either recombinant or derived from human tissues. This defined medium supports long-term culturing/routine culturing of normal cells and of cancer cells, and can be used for freezing and thawing of cells, *i.e*. for cell banking. Here, we show for our defined medium, growth curves and dose response curves of cells grown in two and three dimensions, and applications such as cell migration. Cell morphology was studied in real time by phase contrast and phase holographic microscopy time-lapse imaging. The cell lines used are human cancer-associated fibroblasts, keratinocytes, breast cancer JIMT-1 and MDA-MB-231 cells, colon cancer CaCo-2 cells, and pancreatic cancer MiaPaCa-2 cells as well as the mouse L929 cell line. In conclusion, we present the composition of a defined medium without animal-derived products which can be used for routine culturing and in experimental settings for normal cells and for cancer cells, *i.e.* our defined medium provides a leap towards a universal animal product free cell culture medium.

## Highlights

• The composition of a defined medium without any animal-derived products is presented.
• The medium supports routine culturing of normal cells and cancer cells in 2D and 3D.
• Growth curve data, dose response data, and various methods to study cell migration using the defined medium are presented.
• The medium implies an important step towards removal of foetal bovine serum from cell culturing.

## Graphical Abstract

**Figure:**
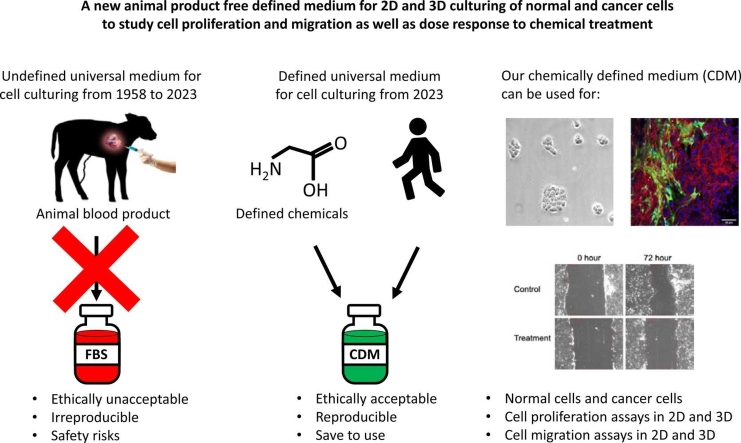

## 1 Introduction

The initiation of culturing of cells outside the body dates back more than 100 years [72]. One of the pioneers was Alexis Carrel who published a series of papers outlining his success with tissue culturing [8], [9], [10], [11]. He did however not have success with long-term culturing because of the hurdle of understanding the composition of medium for cell culturing. The discovery of the nutritional demands of cells resulted in the development of many of the basal media used today, such as RPMI-1640, Ham´s F12, Eagle´s minimum essential medium (MEM), and Dulbecco's modified MEM (DMEM) [72]. The real break-through came in 1958, when Theodore Puck used foetal bovine serum (FBS) (also known as foetal calf serum, FCS) for long-term culturing of cells [50]. Interestingly, Puck et al. observed that FBS collected during different parts of the year supported cell proliferation to different degrees *i.e*. the first report of batch-to-batch variation [50]. Jan van der Valk [63] recently pointed this out, including that Puck et al. had also already mentioned that FBS can contain toxic components which affect the quality of the results [50]. Nevertheless, FBS has remained the most common medium supplement to support cell culturing for 65 years. We still have the scientific problem of batch-to-batch variation concerning FBS and several studies require testing of different batches to obtain optimal results [64]. The issue of reproducibility problems in cell culturing is gaining increased attention [24], [3], [39], [63]. In house results are quite reproducible, especially when using the same batch of FBS, but there is an increasing awareness regarding difficulties in repeating experiments between labs. There are of course many causes, but one of the most mentioned is supplementing 10 % of the cell culture medium with a product, which is an undefined mixture of macromolecules *i.e.* FBS [33], [64]. A seldom mentioned scientific problem with FBS is that it disguises faults we humans make in cell culturing. Those faults are related to routine handling of cell cultures and the faults may be the same within a laboratory but may differ between laboratories. As science becomes more and more exact, we need to move away from undefined products such as FBS. In addition, when studying human diseases or model tissue specific toxicity, we need to use human relevant model systems.

Another serious problem with FBS is the ethical issue surrounding the collection of blood by heart puncture from calf foetuses extracted from the wombs of slaughtered cows [33]. Thus, from an ethical standpoint, a continuing use and production of FBS cannot be justified, as replacements do exist and are continued to be developed [21], [66], [67], [65].

Suggestions for serum replacements can be found in the “fetal calf serum-free database” (fcs-free.org) and in published data [68]. However, more work is needed regarding open publication of known media compositions that support cell culturing. Here, we describe the composition of a medium without any animal-derived products based on compiled knowledge of many years of cell culturing in research and teaching while reading numerous articles mentioning different components of FBS and also in human serum. We have used the medium in different experimental setups. The medium supports the long-term cultivation of cancer cells and normal cells *i.e.* routine passaging. We show data from the use of the medium in different experimental settings of cells grown in two dimensions (2D) and three dimensions (3D). We hope to flatten the hurdle of understanding the composition of medium for cell culturing which has burdened cellular science for so long.

## 2 Materials and methods

### 2.1 Cell lines

The human breast cancer cell line JIMT-1 (ACC589) was purchased from the German Collection of Microorganisms and Cell Cultures, Braunschweig, Germany. The human pancreatic cancer cell line MiaPaCa-2 (CRL-1420™), the human breast cancer cell line MDA-MB-231 (HTB-26™), the human CaCo-2 colon cancer cell line (HTB-37™), and mouse L929 cells (CCL-1™) were purchased from American Tissue Cell Culture, Manassas, VA, USA. Before adaptation to the defined medium, JIMT-1, MiaPaCa-2, and L929 cells were cultured in DMEM/Ham´s F-12 supplemented with 5 % heat-inactivated donor herd horse serum (hi-DHHS) (Sigma-Aldrich Sweden AB, Stockholm, Sweden), 10 μg/ml insulin (Sigma-Aldrich Sweden AB), 20 ng/ml epidermal growth factor (Lonza, Basel, Switzerland), 0.25 µg/ml hydrocortisone (Sigma-Aldrich Sweden AB), 0.2 mM Na-pyruvate, 0.05 mg/ml transferrin (Sigma-Aldrich Sweden AB), 2 mM L-glutamine (Avantor, Radnor, Pennsylvania., USA), 0.1 mM non-essential amino acids (Avantor), 100 μg/ml streptomycin (Avantor), and 100 U/ml penicillin (Avantor). The MDA-MB-231 and CaCo-2 cells were cultured in DMEM/Ham´s F-12 supplemented with 10 % heat-inactivated FBS (Avantor), 0.1 mM non-essential amino acids, 2 mM L-glutamine, 100 μg/ml streptomycin, and 100 U/ml penicillin. Human cancer-associated fibroblasts (CAFs) [40] were obtained from Associate professor Chris Madsen, Medicon Village, Lund University with permission to use from Prof. Akira Orimo, Graduate School Juntendo University, Tokyo, Japan. When obtained, these cells were cultured in DMEM supplemented with 10 % FBS and antibiotics. The human keratinocytes (KeratinoSens cells, suggested for OECD test guideline 442D) were purchased from acCCELLerate, Hamburg, Germany. They were seeded directly into the defined medium described below. The cell lines were kept in an incubator at 37 °C with 5 % CO_2_ in humidified air. Accutase™ (Stem Cell Technologies, Grenoble, France) was used for cell detachment. All cell counting was done manually using a haemocytometer. The cells were passaged once or twice a week depending on seeding density. Cell seeding was always based on cell number. The cells were tested for mycoplasma (Eurofins, Edersberg, Germany) at regular intervals and at the end of all experimental series. No mycoplasma was detected. The adaptation to the defined medium is described below.

### 2.2 The defined medium and tissue culture plastic

The composition of the defined medium is shown in Table 1. The compounds were stored as aliquots between 5 and 250 μl at −80 °C or −20 °C that yielded the final concentration shown in Table 1 after addition to 500 ml of DMEM/Ham´s F12. Depending on solubility, the compounds were dissolved in water, Ca^2+^ and Mg^2+^ free phosphate-buffered saline (PBS), 100 % dimethyl sulfoxide (DMSO), 99.6 % ethanol, or 0.6 M sodium hydroxide. The final concentration of the solutes (DMSO and ethanol) was 0.01 % or below. The pH was not affected by the small volume of 0.6 M NaOH added.

**Table 1:** Composition of the defined medium.

Medium component^a^	Concentration in medium
Basal medium: DMEM / Ham´s F12	**-**
Optional extra buffer: HEPES	10 mM
**Non-proteins**	
All-trans retinoic acid	25 ng/ml
alpha-tocopherol phosphate	3 ng/ml
para-Aminobenzoic acid	12 ng/ml
Ascorbic acid	12 ng/ml
Cholesterol	50 ng/ml
Choline chloride	3.5 μg/ml
Ergocalciferol	25 ng/ml
17-beta Estradiol	0.5 pg/ml
Folic acid	0.33 μg/ml
Glutamine	2 mM
Glutathione	12 ng/ml
Hydrocortisone	0.25 ng/ml
Hypoxanthine Na	1.75 μg/ml
I-inositol	4.5 μg/ml
Linoleic acid	1 μg/ml
Lipoic acid	50 ng/ml
Non-essential amino acids^b^	0.1 mM
*0*-Phosphoryl ethanolamine	5 μg/ml
Pyruvate Na	1 mM
Ribose	125 ng/ml
Selenous acid	8 ng/ml
Thiamine HCl	80 ng/ml
Triiodothyronine	0.2 pg/ml
Uracil	75 ng/ml
Vitamin B12	0.35 μg/ml
Xanthine	85 ng/ml
**Proteins (human)**	
Basic fibroblast growth factor	1 ng/ml
Collagen	100 ng/ml
Epidermal growth factor	10 ng/ml
Fetuin	40 ng/ml
Fibronectin	1.33 μg/ml
Insulin	2 μg/ml
Insulin-like growth factor 1	5 ng/ml
Laminin	20 ng/ml
Platelet-derived growth factor AA	2 ng/ml
Transferrin	50 μg/ml
Vitronectin	100 ng/ml
Human serum albumin	1.25 mg/ml

a The sources and catalogue numbers are found in Supplemental information Table S1.

b L-Glycine, L-alanine, L-asparagine, L-aspartic acid, L-glutamic acid, L-proline, and L-serine.

The cells were cultured in Corning Primaria® (Corning, New York, USA) (cancer cell lines and CAFs) or in Nunclon™ Delta (Roskilde, Denmark) (L929 cells and keratinocytes) tissue culture plastic. All work was performed with the light in the laminar flow bench turned off since the medium is light sensitive.

### 2.3 Adaptation

The CAFs, the L929 cells, and keratinocytes did not need adaptation since they grew immediately when transferred to the defined medium. The JIMT-1, MiaPaCa-2, and CaCo-2 cells went through a 1-month adaption period when the hi-DHHS-supplemented medium was gradually diluted with the defined medium until the cells were cultured in only defined medium. The MDA-MB-231 cells were first cultured in the defined medium with 1 % hi-FBS for two weeks and then transferred to the defined medium. The MDA-MB-231 have so far been passaged 10 times in the medium. MiaPaCa-2 and JIMT-1 have been cultured in the defined medium for 4 years (different batches) and have been authenticated by STR profiling (Eurofins). The L929 cells, the keratinocytes, and CAFs have a stable and specific morphology and growth pattern. The CaCo-2 cells have been cultured for up to 18 passages in the defined medium.

### 2.4 Detachment procedure for cells routine-passaged in defined medium and medium change

At routine passaging, the cell layer was rinsed with PBS. After aspiration of PBS, Accutase™ was added (4 ml/75 cm^2^) and the flask incubated at 37 °C for 10–15 min. Cell detachment was confirmed by looking at the culture in a phase contrast microscope. Then, 5 ml of stopping medium (DMEM/Ham´s F-12 containing human serum albumin (1.25 mg/ml) and antibiotics) was added and the cells were dispersed by careful trituration. Then, 5 ml more of this medium was added and the entire solution with cells was transferred to a sterile 15 ml tube. The cells were pelleted by centrifugation at 300 *g* for 5 min at 4 °C. The medium was removed, and the cell pellet resuspended in the defined medium before cell counting in a haemocytometer. According to our experience, Accutase™ containing medium has to be removed and replaced with fresh defined medium to enable fast cell attachment when reseeding cells. Accutase™ is an animal-derived product as it originates from crustaceans [1]. While this manuscript was under review, we introduced the use of Gibco TrypLE™ (ThermoFisher Scientific), an animal origin free recombinant trypsin-like enzyme and it is fully compatible with the defined medium, thus now we have omitted the use of Accutase™.

When cells were cultured for 1 week, the medium was changed once in between passaging. The replacement medium, did not contain fibronectin.

### 2.5 Freezing and thawing

The cells were frozen in DMEM/Ham´s F-12 supplemented with 10 % human AB serum (Sigma-Aldrich Sweden AB) and 10 % DMSO. The cryovials with 2 × 10^6^ cells were placed in a Corning® CoolCell™ LX Cell Freezing Container, which was put in a −80 °C freezer. Within one week after storing in the −80 °C freezer, the cryovials were transferred to a liquid nitrogen tank.

When thawing, the cryotube was transferred directly from liquid nitrogen to a 37 °C water bath. After two min of thawing, the cell suspension was pipetted to a sterile 50 ml tube. Then, 10 ml of defined medium was added slowly at a rate of 1 ml/min. Defined medium was then added up to 40 ml. The cells were pelleted by centrifugation at 300 *g* at 4 °C for 5 min. The old medium was aspirated, and the cells suspended in 5–10 ml defined medium before cell counting in a haemocytometer. The cells were seeded at a density of 15,000–40,000 cells per cm^2^ with 0.2 ml defined medium per cm^2^. The cells have been frozen and thawed on several occasions.

### 2.6 Growth curve experiments

JIMT-1 and MiaPaCa-2 cells used for growth curve experiments were seeded in Primaria® Petri dishes with a diameter of 3 cm. Both cell lines were seeded at a density of 32000 cells/cm^2^. L929 cells and keratinocytes were seeded in Nunclon™ Delta Petri dishes (diameter of 5 cm) at a density of 19000 and 15000 cells/cm^2^, respectively. At 1, 2, 3, and 4 days after seeding, Petri dishes were removed for cell counting in a haemocytometer after detachment using Accutase™.

### 2.7 Dose response testing in 2D and 3D

In 2D dose response testing using salinomycin, JIMT-1 cells and MiaPaCa-2 cells were seeded as described in Malakpour-Permlid and Oredsson [42] using Primaria® 96-well plates. Keratinocytes and L929 cells were seeded in 2D at a density of 7000 and 5000 cells per well in 180 μl of defined medium in Nunclon™ Delta 96-well plates. When comparing dose response testing in 2D and 3D, JIMT-1 and MiaPaCa-2 cells were cultured in 2D (Primaria® 96-well plates) and 3D (polycaprolactone (PCL)-based 96-well plates, Malakpour-Permlid and Oredsson [42]) as described in Malakpour-Permlid and Oredsson [42] using the same compounds *i.e.* paclitaxel (Tocris Bioscience, Abingdon, United Kingdom) and the salinomycin analogue 20-ethyl carbonate-Na (SAEC). Salinomycin and SAEC were kindly provided by Björn Borgström and Daniel Strand at the Centre for Analysis and Synthesis, Department of Chemistry, Lund University [7]. Stock solutions (10 mM) of salinomycin and SAEC were made in 100 % dimethyl sulfoxide (DMSO), which were stored at −20 °C. A 1 mM stock solution of paclitaxel was made in 100 % DMSO, which was stored at −20 °C. Working solutions were made by dilution in PBS.

### 2.8 Co-culturing of JIMT-1 cells and CAFs in 3D

JIMT-1 cells and CAFs were seeded alone or together in defined medium in the 3D fibre mesh structure as described in Malakpour-Permlid and Oredsson [42]. After 10 days of culturing, the cultures were fixed in 4 % formaldehyde in PBS and then stained with Alexa Flour™ 594 phalloidin (ThermoFisher Scientific, Waltham, MA, USA) and 4′,6-diamidino-2-phenylindole (DAPI) (1 μg/ml PBS) (ThermoFisher Scientific) as described in Pierzynowska et al., [49]. The CAFs express green fluorescent protein [40]. Samples were stored at 4 °C and protected from light before microscopy. Confocal images were taken with a Leica SP8 DLS inverted confocal laser scanning microscope using an oil immersion 20 × /0.75 IMM oil immersion objective lens (Leica Microsystems, Wetzlar, Germany).

### 2.9 Time-lapse videos of general cell movement

For time-lapse imaging, two different microscopes were used: a HoloMonitor® M3 phase holographic microscope (M3) (PHI AB, Lund, Sweden), with a 10 times phase contrast objective, placed in an incubator at 37 °C with air as atmosphere and a HoloMonitor® M4 phase holographic microscope (M4) (PHI AB) placed in a regular 37 °C CO_2_ incubator with 5 % CO_2_ in air. For time-lapse imaging of routine cultures in the M3 microscope, the cells were seeded in a 25 cm^2^ Primaria® tissue culture flask, which was incubated for 24 h in a 37 °C CO_2_ incubator. When removed from the incubator, the lid of the flask was closed tightly to preserve the 5 % CO_2_ in air atmosphere and the flask was placed on the stage of the M3 microscope. Images were captured every 10 min for 72 h. For time-lapse imaging of cells in the M4 microscope, cells were seeded in 6-well Primaria® plates. The cells were allowed to attach for 24 h prior to initiation of the time-lapse imaging. The standard lid of the 6-well plate was replaced with three HoloLid® 71120 for 6-well plates (PHI AB). The 6-well plate was then placed on the motorised stage of the M4. For imaging in the M3 and M4 microscopes, the software programmes Hstudio® (PHI AB) and HoloMonitor® Appsuite (PHI AB) were used, respectively. In the M4 microscope, images were acquired at 6–8 locations per well, at 5 min intervals for 72 h. Only one location was imaged in the M3 microscope.

### 2.10 Cell movement from spheroids

Movement of cells from spheroids was analysed using the M3 and M4 microscopes. Spheroids seeded in Primaria® 6-well plates were analysed using the M4 microscope while spheroids seeded on linear fibres of PCL were analysed in the M3 microscope.

Cancer cells alone (100000 JIMT-1 or 50000 MiaPaCa-2 cells) or cancer cells (100000 JIMT-1 or 50000 MiaPaCa-2 cells) together with CAFs (100000) were seeded in hydrophobic Petri dishes (diameter 3 cm, 1.5 ml defined medium) and the cultures incubated for 5 days in a 37 °C CO_2_ incubator on a rotating shaker table. The MiaPaCa-2 cells formed tight spheroids while the JIMT-1 spheroids were irregular and loose. The MiaPaCa-2/CAF spheroids were even denser than MiaPaCa-2 spheroids alone. The JIMT-1/CAF spheroids were irregular. We did not optimise spheroid size as we were interested in cell migration from the spheroid edges in the defined medium. In general, several spheroids were formed, and they were of similar size.

For analysis in the M4 microscope, the spheroids were added to 6-well Primaria® plates. Before adding the spheroids, a small area (0.5 cm^2^) in the centre of each well was incubated with a small drop of medium for 1 h. The spheroids were added to the drop with a micropipette. To prevent drying out, 1 ml of medium was added along the wall of each well. Capillary action prevented the medium from flowing to the drop. After 24 h of incubation, medium was added to a final volume of 4 ml. The standard lid of the Petri dish was replaced with three HoloLid® 71120 for 6-well plates. Spheroids were also subjected to treatment with 100 nM paclitaxel. The 6-well plate was then placed on the motorised stage of the M4 microscope. The edge of the spheroid to be imaged was kept in focus at the edge of the viewing field using the application Kinetic Motility Assay in HoloMonitor® App Suite and imaging every 5 min for 72 h was initiated. The Wound Healing application in HoloMonitor® App Suite was used to analyse cell movement from the spheroids. After the 72 h of imaging, the samples were fixed in 4 % formaldehyde in PBS followed by staining with Alexa Flour™ phalloidin 488 (ThermoFisher Scientific) to visualise the actin filaments by immunofluorescence microscopy.

For analysis in the M3 microscope, spheroids were seeded on linear fibres of biocompatible PCL [43]. Linear fibres of PCL were obtained as described in Malakpour-Permlid et al. [43] with the difference that a rotating collector was used, and the time of electrospinning was carefully controlled to obtain a single layer of parallel fibres. The fibres were spun on a plastic backing and this was cut into 1 cm^2^ pieces. The fibres were subjected to plasma treatment as described in Malakpour-Permlid et al. [43] and they were then sterilised in 70 % ethanol before seeding of spheroids. One to two spheroids were seeded per plastic substrate with parallel fibres. After 24 h of incubation, the piece was carefully transferred to a 25 cm^2^ regular cell culture flask and the flask was returned to the incubator for 2 h to equilibrate the atmosphere in the flask to 5 % CO_2_. Then, the lid was tightened, and the flask moved to the stage of the M3 microscope. Images were captured every 10 min for 72 h. After the 72 h of imaging, the samples were fixed in 4 % formaldehyde in PBS followed by staining with Alexa Flour™ phalloidin 488 (ThermoFisher Scientific) and DAPI to visualise the actin filaments and cell nuclei, respectively, by immunofluorescence microscopy.

### 2.11 Scratch wound healing assay

The keratinocytes were used in a conventional scratch wound healing assay. For this assay, 1.9 × 10^6^ keratinocytes were seeded in 2 ml of defined medium in Petri dishes (Nunclon™ Delta) with a diameter of 3 cm. After 24 h of incubation, three scrape wounds were made using a sterile pipette tip. The plates were rinsed 3 times with warm PBS and then 2 ml of defined medium without any growth factors was added. Growth factors were removed to reduce the effect of cell proliferation. Salinomycin was added to a final concentration of 0.25 μM. The scrape wounds were imaged using a phase contrast microscope (Olympus CKX41 equipped with a XM10 camera, Olympus Sverige AB, Solna, Sweden) and the Petri dishes returned to the CO_2_ incubator. After 72 h of incubation, the scrape wounds were imaged again. The size of the wounds was analysed using the software cellSens Dimensions (Olympus Sverige AB).

### 2.12 Statistical analysis

The data are presented as mean ± SD of at least three independent samples or experiments, and was analysed using GraphPad Prism 9, version 9.4.1 (GraphPad Software Inc., San Diego, California, USA). When applicable, ordinary one-way ANOVA using multiple comparisons was used to elucidate statistical differences in the obtained data. When two groups were compared, Student´s t-test was used.

## 3 Results

### 3.1 Growth curves

**Fig. 1:**
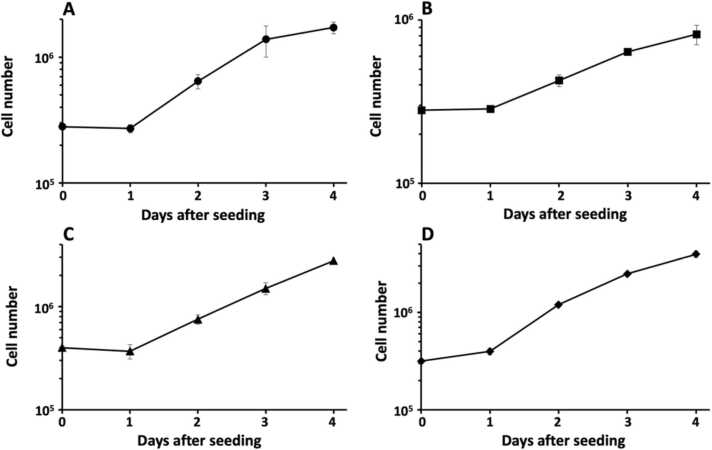
Growth curves of human pancreatic cancer MiaPaCa-2 cells (A), human breast cancer JIMT-1 cells (B), L929 mouse fibroblasts (C), and human keratinocytes (D) cultured in the defined medium. The data for each cell line is compiled from 2 to 5 different experiments (where the number of replicates in each experiment is 3) performed by different persons with different passage numbers of the cells. The error bars show ± SEM.

One of the basic assays employed to analyse cell proliferation dynamics of cultured cells is to study their growth curve. Thus, we established growth curves for MiaPaCa-2 cells, JIMT-1 cells, L929 fibroblasts, and keratinocytes in the defined medium (Fig. 1).

The growth curves show a distinct lag phase before commencement of exponential cell proliferation. The population doubling time during the exponential growth phase was 20, 41, and 24 h for MiaPaCa-2 cells, JIMT-1 cells, and L929 fibroblasts, respectively. The population doubling time was 15 h and 23 h for the keratinocytes between days 1 and 2 and days 2 and 3, respectively. We believe the keratinocytes were partially synchronised on day 1 after seeding and therefore massive cell division took place between days 1 and 2 resulting in the short population doubling time before the cell population was distributed in the cell cycle. Two different experiments showed the same proliferation pattern *i.e*. a large increase in cell number between days 1 and 2 after seeding compared to days 2 and 3. Table 2 shows population doubling times obtained in the laboratory for the used cell lines cultured in the defined medium, medium supplemented with 10 % hi-FBS, or with 5 % hi-DHHS, for comparison.

**Table 2:** Population doubling times obtained in the laboratory for the used cell lines cultured in the defined medium, medium supplemented with 10 % hi-FBS, or with 5 % hi-DHHS.

Cell line	Medium	Ref	PDT^a^ (hours)
**MiaPaCa-2**	Defined medium	Present data	20
	10 % hi-FBS	Not published^b^	≈ 25
	10 % hi-FBS	[58]	21
	5 % hi-DHHS	Not published^b^	≈ 26
**JIMT-1**	Defined medium	Present data	41
	10 % hi-FBS	[41]	23
	10 % hi-FBS	[37]	22
	10 % hi-FBS	[56]	20
	5 % hi-DHHS	Not published^b^	≈ 28
**L929**	Defined medium	Present data	24
	10 % hi-FBS	Not published^b^	≈ 22
	5 % hi-DHHS	[37]	21
**Keratinocytes**	Defined medium	Present data	15, 23

a PDT, population doubling time.

b Not published, data obtained by students in BIOR21 (Toxicology) and BIMM18 (Cell Culturing) courses at the Department of Biology, Lund University, Sweden during 2015–2021.

### 3.2 Dose response testing

**Fig. 2:**
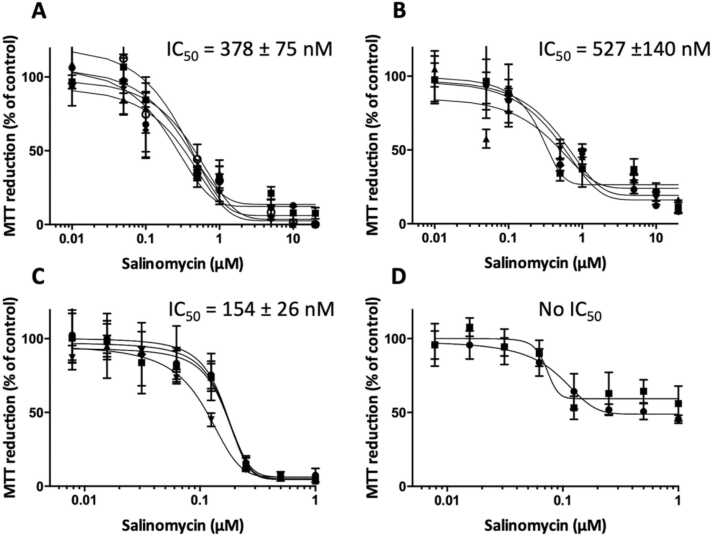
Dose response curves for salinomycin-treated MiaPaCa-2 cells (A), JIMT-1 cells (B), L929 cells (C), and keratinocytes (D) cultured in the defined medium. The cells were seeded and incubated for 24 h to allow attachment before addition of compound at the indicated concentrations. After 72 h of incubation, the toxicity was evaluated using an MTT assay. The curves are drawn in GraphPad Prism 9. Each curve represents an independent experiment. Each data point is the mean ± SD of 6 wells. The IC_50_ values are the mean ± SD for all experiments for each cell line.

The most common means to get an understanding of toxicity of a compound is to perform dose response tests and therefore it is imperative that the defined medium can be used for such tests. Fig. 2 shows dose response curves for MiaPaCa-2 cells (Fig. 2A), JIMT-1 cells (Fig. 2B), and L929 mouse fibroblasts (Fig. 2C), and keratinocytes (Fig. 2D) grown in 2D in the defined medium treated with salinomycin. Salinomycin is a compound initially discovered to target cancer stem cells [23]. Through selective chemical modification of salinomycin, a library of analogues was obtained, and we showed improved selectivity towards cancer stem cells, specifically the compound SAEC [25], [26], [27], [7]. Half maximal inhibitory concentration (IC_50_) values (seen in Fig. 2) were found for all cell lines cultured in 2D treated with salinomycin, except for the keratinocytes.

Table 3 shows IC_50_ values for salinomycin-treated cells cultured in our laboratory in different media for comparison.

**Table 3:** IC_50_ values obtained in dose response experiments obtained in the laboratory for the used cell lines treated with salinomycin cultured in the defined medium, in medium supplemented with 10 % hi-FBS, or with 5 % hi-DHHS.

Cell line	Medium	Reference	IC_50_ (nM)
MiaPaCa-2	Defined medium	Present data	378 ± 75
	10 % hi-FBS	Not published^a^	≈ 250
	5 % hi-DHHS		No data
JIMT-1	Defined medium	Present data	527 ± 140
	10 % hi-FBS	[7]	520 ± 90
	10 % hi-FBS	[25]	380 ± 30
	5 % hi-DHHS	Not published^a^	240 ± 7
L929	Defined medium	Present data	154 ± 26
	10 % hi-FBS		No data
	5 % hi-DHHS	Not published^a^	197 ± 50
Keratinocytes	Defined medium	Present data	No IC_50_

a Not published, data obtained by students in BIOR21 (Toxicology) and BIMM18 (Cell Culturing) courses at the Department of Biology, Lund University, Sweden during 2015–2021.

**Fig. 3:**
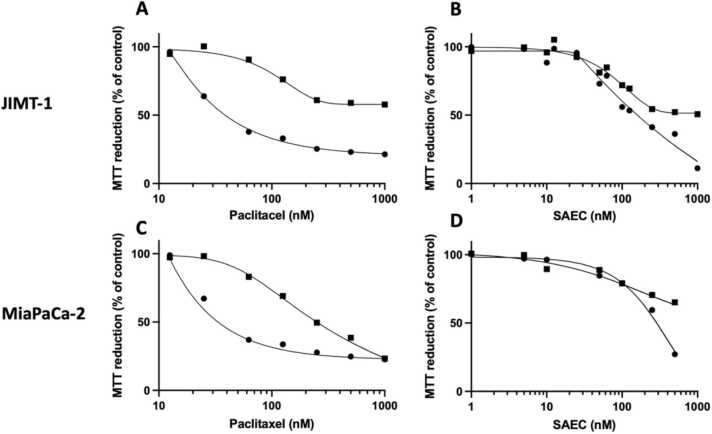
Dose response curves for JIMT-1 human breast cancer cells (A and B) and MiaPaCa-2 pancreatic cancer cells (C and D) in the defined medium in 2D cultures (closed circles) and 3D cultures (closed squares) treated with paclitaxel or SAEC. The cells were seeded and incubated for 24 h to allow attachment before addition of compound at the indicated concentrations. After 72 h of incubation, the toxicity was evaluated using an MTT assay. The curves are drawn in GraphPad Prism 9 using all data from three independent experiments for each curve. Individual dose response curves for each of the three experiments are found in Supplementary Figs. S1 and S2.

In a previously published paper [42], we compared the toxicities of paclitaxel and SAEC, a synthetic salinomycin analogue [26], [7], in JIMT-1 cells and human dermal fibroblasts grown in 2D and 3D in a medium supplemented with 5 % hi-DHHS. Here we investigate the toxicity of paclitaxel and SAEC in JIMT-1 and MiaPaCa-2 cells grown in 2D and 3D (Fig. 3) in the defined medium.

As we found previously for cells grown in medium supplemented with 5 % hi-DHHS [42], the IC_50_ values were higher for cells treated with paclitaxel or SAEC when cultured in the defined medium in 3D compared to 2D (Table 4).

**Table 4:** IC_50_ values in nM concentrations obtained after treating JIMT-1 and MiaPaCa-2 cells cultured in 2D and 3D in the defined medium.

	JIMT-1		MiaPaCa-2	
	**2D**	**3D**	**2D**	**3D**
**Paclitaxel (nM)**	41.9 ± 3.0^a^	NA^b^	38.8 ± 11.7^c^	274.5 ± 106.4
**SAEC (nM)**	139.2 ± 38.1	NA	286.7 ± 13.8	NA

a Mean ± SD of three independent experiments.

b NA, not applicable within the dose range studied.

c Student´s t-test comparing 2D and 3D, p < 0.02.

### 3.3 Confocal microscopy of 3D-cultured cells

**Fig. 4:**
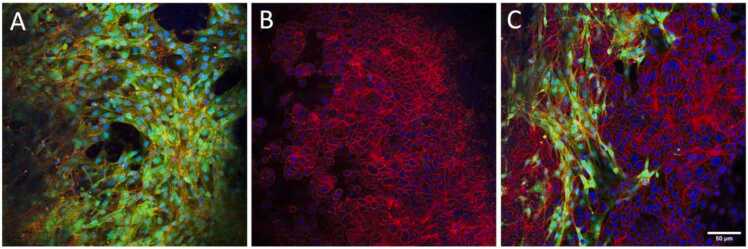
Single confocal microscopy plane images of CAFs (A), JIMT-1 cancer cells (B), and a co-culture of JIMT-1 cells and CAFs (C) cultured in a 3D network of PCL fibres in defined medium for 10 days. After fixation in 4 % formaldehyde in PBS, the cultures were stained with Alexa Flour™ 594 phalloidin (actin filaments, red) and DAPI (cell nuclei, blue). The CAFs express green fluorescent protein [40] and thus the actin filaments are orange in the CAFs due to colour overlap. All images are representative of at least three different experiments. Scale bar indicates 50 µm.

We have previous shown that JIMT-1 cells and human dermal fibroblasts grown in medium supplemented with 5 % hi-DHHS as mono-cultures and as a co-culture grow throughout the depth of the same kind of 3D PCL-based structures as used here [42]. Here we show that CAFs, JIMT-1, and both cell lines together grow in 3D in the defined medium in a similar manner as we have shown previously (Fig. 3). The images show a single confocal microscopy plane in the middle of the cultures. In co-culture, the JIMT-1 cells grow in tight clusters surrounded by the CAFs. Fig. 4.

### 3.4 Time-lapse imaging

In general, cells are only viewed statically in a phase contrast microscope and therefore information about cell behaviour in between microscopic evaluation is less known. We have studied cell behaviour during several days by time-lapse imaging in M3 or M4 holographic microscopes (Table 5 lists the videos shown in Supplemental information). Supplemental Video S1 shows an M3-derived phase contrast microscope time-lapse video of keratinocytes dancing in defined medium (real time 72 h). Single keratinocytes and keratinocytes in groups show circular movement where they appear to search for each other and make larger groups of cells. We also have videos from the M4 microscope showing the same behaviour (data not shown). The Supplemental Video S2 shows a phase holographic time-lapse video of CaCo-2 cells in defined medium (real time 72 h). The CaCo-2 cells do not move around substantially. Several cell divisions are seen. The CaCo-2 cells are not optimal for phase holographic imaging as they are very thin, and the entire cells are actually not seen. Phase contrast microscopy shows that the cells form a tight sheet and each cell has a rather large cytoplasm (supplemental Figure S2, videos from the M3 microscope show the same behaviour (data not shown)). Holographic microscopy shows the thicker parts of cells *i.e.* the cell nuclei and cytoplasm close to the nuclei, but not the very thin parts of the cytoplasm. This is a well-known phenomenon related to phase holographic imaging, since the phase shift of the light in very thin parts of a cell is too small to create an interference pattern or hologram (www.phiab.com). Supplemental Video S3 (phase holographic time-lapse) shows well-defined elongated MDA-MB-231 cells moving around at high speed, and several divisions where the cells round up are seen. Supplemental Video S4 (phase holographic time-lapse) shows well-defined JIMT-1 cells. All these videos show the behaviour of cells in routine passage.

**Table 5:** Time-laps videos shown in the supplemental information.

Supplemental Video Number	Featuring
S1	Phase contrast microscopy time-lapse video of keratinocytes cultured in the defined medium.
S2	Phase holographic microscopy time-lapse video of human colon cancer CaCo-2 cells cultured in the defined medium.
S3	Phase holographic microscopy time-lapse video of human breast cancer MDA-MB-231 cells cultured in the defined medium.
S4	Phase holographic microscopy time-lapse video of human breast cancer JIMT-1 cells cultured in the defined medium.
S5	Phase holographic microscopy time-lapse video of human breast cancer JIMT-1 spheroids cultured in the defined medium.
S6	Phase holographic microscopy time-lapse video of human pancreatic cancer MiaPaCa-2 spheroids cultured in the defined medium.
S7	Phase holographic microscopy time-lapse video of human breast cancer JIMT-1/CAF spheroids cultured in the defined medium.
S8	Phase holographic microscopy time-lapse video of human pancreatic cancer MiaPaCa-2/CAF spheroids cultured in the defined medium.
S9	Phase holographic microscopy time-lapse video of paclitaxel-treated (100 nM) human breast cancer JIMT-1 spheroids cultured in the defined medium.
S10	Phase holographic microscopy time-lapse video of paclitaxel-treated (100 nM) human breast cancer JIMT-1/CAF spheroids cultured in the defined medium.
S11	Phase contrast microscopy time-lapse video of human breast cancer JIMT-1 spheroids seeded on parallel PCL fibres in the defined medium.
S12	Phase contrast microscopy time-lapse video of human pancreatic cancer MiaPaCa-2 spheroids seeded on parallel PCL fibres in the defined medium.

**Fig. 5:**
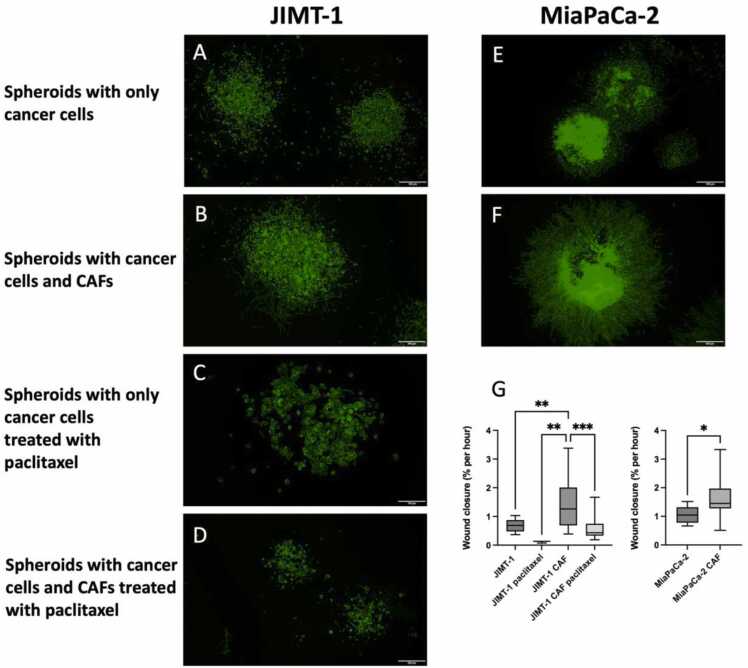
Spheroids of JIMT-1 cells (A) and MiaPaCa-2 cells (E) alone or in the presence of CAFs (B and F, respectively) cultured on a flat surface in the defined medium after 3 days of imaging in a phase holographic microscope. C and D shows JIMT-1 spheroids and JIMT-1/CAF spheroids, respectively, treated with 100 nM paclitaxel. After the 3 days of time-lapse imaging in the M4 microscope, the spheroids were fixed in 4 % formaldehyde in PBS and stained with Alexa Flour™ phalloidin 488 to visualise the actin filaments by immunofluorescence microscopy. The scale bars are 500 µm in all images except in C where the scale bar is 100 µm. G shows the rate of migration of cells from spheroid edges deduced from the time-lapse videos using the Wound Healing Application in the software programme HoloMonitor App Suite (representative videos for the treatments: Supplemental Videos S5-S10). The data show the mean and min/max values. Number of experiments for spheroids with JIMT-1 cells: values from n ≥ 13 independent videos, except for paclitaxel-treated JIMT-1 spheroids where n = 3 videos. The JIMT-1 spheroids were extremely sensitive to paclitaxel treatment, explaining the few samples. Number of experiments for spheroids with MiaPaCa-2 cells: values from n ≥ 9 independent videos.

We have used the M4 to study the movement of cells from spheroids seeded on a flat surface. We were interested in investigating if cell movement was affected by the presence of CAFs which has been demonstrated by others using medium supplemented with 10 % FBS [16], [47], [61] and we have also treated spheroids with paclitaxel. The behaviour of cells in JIMT-1 spheroids (Supplemental Video S5), in MiaPaCa-2 spheroids (Supplemental Video S6), in JIMT-1/CAF spheroids (Supplemental Video S7), and in MiaPaCa-2/CAF spheroids (Supplemental Video S8) was investigated with the M4 microscope. Spheroids of JIMT-1 cells alone and JIMT-1/CAF spheroids were also treated with 100 nM paclitaxel (Supplemental Videos S9 and S10, respectively). The representative Supplemental Videos S5-S10, show the movement of cells from the spheroid edge into the viewing field for 72 h (except Supplemental Video S8, 48 h). The videos show that the MiaPaCa-2 cells move faster than the JIMT-1 cells (compare Supplemental Videos S5 and S6). When CAFs were present, cell movement increased (compare Supplemental Videos S5 and S6 with Supplemental Videos S7 and S8, respectively). Paclitaxel treatment impaired cell movement as can be seen in Supplemental Videos S9 and S10. When the imaging was finished (after 72 h of culturing), the spheroids were fixed in 4 % formaldehyde in PBS and stained for immunofluorescence microscopy (Fig. 5). Fig. 5A and 5B show how JIMT-1 and MiaPaCa-2 cells, respectively, have migrated radially from a central core. The JIMT-1 spheroids were less compact than the MiaPaCa-2 spheroids. When co-spheroids of cancer cells and CAFs were seeded, the fibroblasts migrated ahead of the cancer cells, however, this was always more obvious from MiaPaCa-2/CAF spheroids compared to JIMT-1/CAF spheroids. Fig. 5C and 5D show spheroids of JIMT-1 cells and JIMT-1/CAFs, respectively, treated with 100 nM paclitaxel. The wound closure rate deduced from several videos using the Wound Healing application in HoloMonitor® App Suite for each treatment is shown in Fig. 5G. When CAFs were present, the rate of wound closure was significantly higher for both JIMT-1 and MiaPaCa-2 spheroids compared to spheroids of the cancer cells alone (Fig. 5G). Paclitaxel treatment significantly reduced the rate of wound closure of JIMT-1 cells alone and JIMT-1/CAFs (Fig. 5G).

### 3.5 Cell migration along parallel fibres

**Fig. 6:**
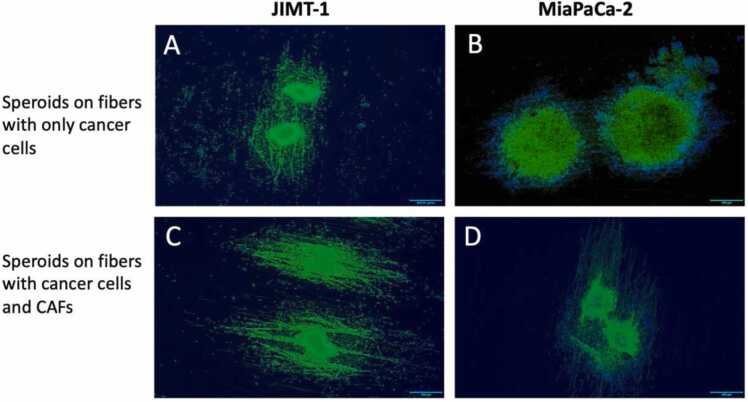
Spheroids of JIMT-1 cells (A) and MiaPaCa-2 cells (B) alone or in the presence of CAFs (C and D, respectively) cultured on parallel PCL fibres in defined medium for 5 days. Then, the spheroids were fixed in 4 % formaldehyde in PBS and stained with Alexa Fluor™ phalloidin 488 to visualise the actin filaments and with DAPI to visualise the nuclei by immunofluorescence microscopy. Scale bars are 500 µm.

Besides seeding spheroids on a flat surface, we seeded them on parallel PCL fibres. The purpose was to investigate if cell movement seen in *intravital* microscopy could be reflected by cell movement in a defined medium. Fig. 6 shows spheroids that have been cultured on the PCL fibres for 5 days. The fibres are spun on a backing, and they become somewhat distorted by the fixation and staining. Instead of radial migration seen when spheroids were seeded on a flat surface, the cells moved along the fibres similar to directional migration of cancer cells from a tumour bulk. Figs. 6A and 6B show spheroids of JIMT-1 and MiaPaCa-2 cells, respectively. Fig. 6A shows dispersed JIMT-1 cells outside of the spheroids reflecting that they were not compact. As mentioned above, the JIMT-1 spheroids were always looser and somewhat more difficult to handle compared to the compact MiaPaCa-2 spheroids. Our notion is that the cells of MiaPaCa-2 spheroids migrated more collectively as a bulk of cells, *i.e.* the entire spheroid expanded while individual cells moved out from the JIMT-1 spheroids. Figs. 6C and 6D show spheroids of JIMT-1 and MiaPaCa-2 cells, respectively containing CAFs. The CAFs appeared to migrate out ahead of the cancer cells.

Supplemental Videos S11 and S12 show M3-derived phase contrast time-lapse videos of JIMT-1 and MiaPaCa-2 cells, respectively, migrating along parallel PCL fibres from a spheroid. Both cell types show collective and amoeboid movement with cells moving back and forth as has been found for cancer cells in *intravital* microscopy [19]. However, there is also a distinct bulk movement of cells from the MiaPaCa-2 spheroid.

Supplementary material related to this article can be found online at doi:10.1016/j.toxrep.2023.04.001.

The following is the Supplementary material related to this article Videos S1, Video S2, Video S3, Video S4, Video S5, Video S6, Video S7, Video S8, Video S9, Video S10, Video S11 and Video S12.

Video S1

Video S2

Video S3

Video S4

Video S5

Video S6

Video S7

Video S8

Video S9

Video S10

Video S11

Video S12

### 3.6 Cell migration in a scratch wound assay

**Fig. 7:**
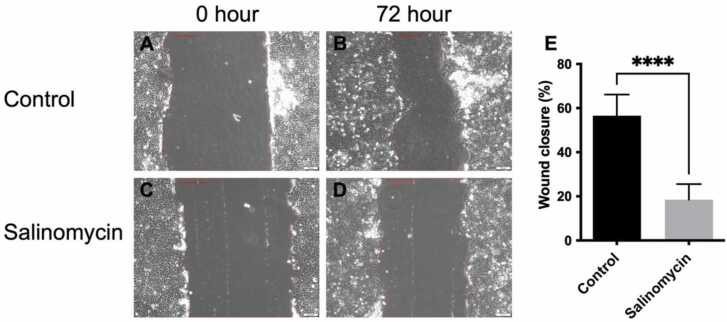
Wound closure by human keratinocytes cultured in defined medium. At time 0 h (A and C) a wound was created, and images taken after addition of salinomycin to a final concentration of 0.25 μM. After 72 h of incubation, images were taken again, and the wound closure calculated. The images are representative of n = 13 independent wounds from 2 independent experiments. The columns show the mean and bars SD. * ** *, P ≤ 0.0001.

Wound healing assays are classical and commonly used methods to study cell migration and the molecular biology underlying it, as well as effect of compounds on the process. We used the human keratinocytes in a scratch wound healing assay with defined medium and compared control with salinomycin-treated cells (Fig. 7). Treatment of the keratinocytes for 72 h with salinomycin significantly reduced the wound healing *i.e.* migration of cells into the wound area.

## 4 Discussion

Although the reproducibility, safety and ethical problems with FBS are widely recognised, FBS is still extensively used in cell culture media [12]. Defined medium has been developed and is used in various cellular models (some examples: [29], [60], [4]; see FCS-free database fcs-free.org) but routine passaging is performed in medium supplemented with FBS. Our approach was to develop a universal defined medium with human proteins that could be used for reproducible routine passaging and for cell banking as well as in different experimental settings. The relevance of the defined medium is proven with several cell types (normal and cancer) cultured in 2D and 3D, using end points such as growth curves, dose response curves, cell migration, and effects of known reference chemicals. Here, we present the composition of the defined medium which instead of serum contains human proteins for cell attachment/spreading and stimulation of cell proliferation. We used this medium in five practical sessions with L929 cells and keratinocytes in the course BIOR21 Toxicology at the Department of Biology, Lund University in the spring of 2022. Twenty-four students performed growth curve experiments, dose response experiments, wound healing experiments, live/dead cell assays, and they also successfully used the keratinocytes in the OECD *in vitro* skin sensitisation assay TG 442D (data not shown).

As a basal medium for the defined medium, we choose DMEM/Ham´s F12 and added the non-protein components found in Ince et al. [31]. However, this is not yet a medium that enables long-term survival. To support long-term routine cell culturing, different protein components have to be added. These are proteins that are carriers, are required for cell attachment and spreading, and for stimulation of cell proliferation. The most important protein carrier in human blood is albumin with a concentration of about 40 mg/ml [17]. In FBS, the albumin concentration is about 50 mg/ml [14]. Most often serum is used at a concentration of 10 % yielding a final albumin concentration of 4–5 mg/ml depending on serum type. We decided to add albumin isolated from human serum to a concentration of 1.25 mg/ml to reduce the protein content to facilitate downstream processing and improve biosafety. The weakest point in the defined medium is the human serum albumin which has > 92 % purity according to the producer Biowest *i.e.* not 100 % as would be desired. Thus, the medium may contain a maximum of 100 ng/ml of unknown compounds. We are well aware of this but still consider this medium superior compared to adding 10 % of serum containing a multitude of unknown factors.

Collagen and fibronectin, the main proteins supporting cell attachment and spreading, are usually used for coating tissue culture plastics in a serum reducing context. We added these components directly to the medium at the concentrations 100 ng/ml (collagen) and 1.33 μg/ml (fibronectin). Collagen and fibronectin in blood are synthesised by hepatocytes and are found at a concentration of around 30–50 ng/ml [38] and 300 μg/ml [20], respectively. Other proteins mentioned in the context of cell attachment and spreading are fetuin, laminin, and vitronectin and thus we decided to add these proteins as well at indicated concentrations according to literature [53].

After having attached and spread out, cells need signals from growth factors to initiate cell proliferation. Epidermal growth factor, a potent mitogen, is commonly added to cell culture media. Another growth factor that regulates cell growth and division is platelet-derived growth factor which is found in a high content in serum due to release from platelets when blood clots [2], [59]. In fact, human platelet lysate (hPL) has been suggested as a replacement for FBS because of the high concentration of growth factors [5], [68]. We also added insulin-like growth factor 1 and basic fibroblast growth factor as these proteins have proven to be part of stimulation of cell proliferation [52].

Two important proteins found in serum that need to be added to cell culture medium are insulin and transferrin. Insulin has a complex role in cell culture medium stimulating cell growth, cell cycle progression, and glucose uptake [71]. Transferrin is a carrier protein of iron, both to facilitate iron uptake but also to protect cells from the potential toxicity of Fe^2+^ that initiates the Fenton reaction [13].

As mentioned above, the basis for the composition of our defined medium is found in a paper published in 2007 by Ince et al. [31]. In that paper, they describe the use of Corning Primaria® tissue culture plastic, which has both positive and negative charges due to the incorporation of nitrogen and oxygen, respectively. Typical cell culture plastic such as Nunclon™ Delta is only negatively charged. Our preference is to use the Corning Primaria® tissue culture plastic, as the natural extracellular matrix is both positively and negatively charged.

The adaptation process was different for each cell line as mentioned in the results section and we considered that the cells were adapted when cell growth was constant determined by routine passaging. Several references with adaptation protocols are found in the [46]. We followed the adaptation protocol presented by van der Valk et al., [62], but we also always tried to culture the cells directly in the defined medium. The first criterion of adaptation was that the cells had attached and spread one day after seeding. The second criterion was that the cell number had increased at the following passage a week later and that this was repeatable for several passages. The cell lines adapted to the defined medium have been frozen and thawed on several occasions. A cell bank based on defined medium has been established for the cell lines used here *i.e.* CAFs, JIMT-1 breast cancer cells, MDA-MB-231 breast cancer cells, MiaPaCa-2 pancreatic cancer cells, CaCo-2 human colon cancer cells, human keratinocytes, and mouse L929 fibroblasts.

When the cells were considered adapted, *i.e*. the routine passaging was stable, we established growth curves for some cell lines as shown in the results section. These growth curves are derived from different batches of the used cells over time and the data obtained by different persons. The population doubling times we found for MiaPaCa-2 cells and L929 cells cultured in the defined medium are close to those we found for the same cell lines cultured in hi-FBS- or hi-DHHS-supplemented medium. JIMT-1 cells grow slower in the defined medium compared to medium supplemented with hi-FBS or hi-DHHS when cultured in our laboratory. Reported population doubling times in FBS-supplemented medium for MiaPaCa-2 cells are 26–40 h (www.cellosaurus.org/CVCL_0428), for JIMT-1 cells 30–40 h (www.cellosaurus.org/CVCL_2077), and for L929 cells 21–28 h (web.expasy.org/cellosaurus/CVCL_042).

After establishing growth curves, we set out to perform 2D dose response assays as they are important in compound screening in the early drug discovery process [28]. For these assays, we used the experimental compound salinomycin, which was discovered to be a selective inhibitor of breast cancer stem cells by Gupta et al. [23]. We have investigated the toxicity and molecular mechanism of salinomycin and a number of salinomycin analogues using medium supplemented with hi-FBS or hi-DHHS (Crichton et al., 2013; [25], [26], [6], [37], [27], [36]). Here we present IC_50_ values obtained from dose response curves for MiaPaCa-2 cells, JIMT-1 cells, and L929 cells cultured in the defined medium, as well as not published and published data from the cell lines cultured in medium supplemented with 10 % hi-FBS or 5 % hi-DHHS. Comparison of the data confirms the validity of the defined medium.

We also compared dose response data obtained from cells in 2D and 3D cultures, because of the awareness that the latter shows better potential in *e.g.* simulating the tumour microenvironment [15], [32], [55]. A number of studies with different cell lines cultured in medium with 10 % FBS have shown a higher toxicity of chemical treatment of cells cultured in 2D compared to 3D (*i.e.* lower IC_50_ in 2D compared to 3D) (Loessner et al., 2010; [45], [30], [57]). We used the well-known chemotherapeutic drug paclitaxel and the experimental salinomycin analogue SAEC to be able to compare with our published data with JIMT-1 cells cultured in medium supplemented with 5 % hi-DHHS [42]. Here we show that JIMT-1 and MiaPaCa-2 cells cultured in the defined medium are more sensitive to paclitaxel and SAEC when grown in 2D compared to 3D. The IC_50_ values for JIMT-1 cells treated with paclitaxel or SAEC are similar to those obtained when JIMT-1 cells were cultured in medium supplemented with 5 % hi-DHHS in 2D and 3D [42]. Thus, these data also confirm the validity of the defined medium.

In our study, spheroids were formed by seeding cancer cells alone or together with CAFs in the defined medium in hydrophobic Petri dishes and incubating them on a rotary shaker placed in a 37 °C CO_2_ incubator. CAFs have been shown to have a supporting role in the tumour microenvironment stimulating cancer cell proliferation, invasion, and metastasis, imparting resistance to cancer therapeutic drugs as well as stimulating angiogenesis [35]. By time-lapse imaging using the M4, we monitored the rate of cell migration from the spheroid edges. Others have shown that CAFs affect the rate of cancer cell migration in model systems using medium supplemented with 10 % FBS [16], [47], [61]. Here we show that CAFs increased the rate of cell migration from spheroids containing CAFs and cancer cells compared to the rate of migration from spheroids with only cancer cells using the defined medium. Furthermore, we show that CAFs modulate the toxicity of paclitaxel. Here, we show proof-of-principle that it is possible to form spheroids with cancer cells only or together with CAFs in the defined medium and to use the spheroids in different assays and applications.

Besides seeding spheroids on flat surfaces, we seeded the them on parallel fibres of PCL which mimic the collagen fibres of the extra cellular matrix. PCL fibres have been extensively used for the study of cell migration *in vitro* in medium supplemented with FBS (Kelleher and Vacanti, 2010; [48], [22], [44], [34]). Using *intravital* microscopy, it has been shown that cancer cell migration follows stromal structures such as aligned collagen [51], [54]. Our notion is that the animal requiring and ethically questionable *intravital* microscopy as well as using animal-derived collagen can be replaced by investigating cell movement along collagen mimicking PCL fibres. As a proof-of-concept, we studied the movement of cancer cells along parallel fibres of PCL using time-lapse phase contrast microscopy. We found that the cells display both collective movement as well as single cell movement along the PCL fibres as has been shown using *intravital* microscopy [18], [19]. Thus, cancer cell movement along parallel PCL fibres can be studied using the defined medium and further studies should be aimed at confirming similar molecular mechanisms as those found in *intravital* microscopy to fully justify the system.

Epidermal keratinocytes together with fibroblasts are important for skin re-epithelialisation during wound healing [70]. There is vast research ongoing regarding wound healing, specifically in the context of chronic wounds using keratinocytes and fibroblasts [69]. In these studies, mainly medium with FBS have been used [12]. Here we show that human keratinocytes cultured in the defined medium can be used in a scratch wound healing assay, thus rendering the use of FBS dispensable in this research area.

## 5 Conclusions

In conclusion, here we present the composition of a medium free of FBS with a defined content of chemicals and human proteins. The defined medium showed to be adequate for routine culturing, including freezing and thawing for a number of different normal and cancer cell lines. In addition, we have shown that the defined medium can be used in different experimental settings such as to obtain growth curves, dose response curves in 2D and 3D, and to study cell migration in different contexts. The validity of the defined medium was confirmed by comparing with data obtained by culturing cells in FBS- or DHHS-supplemented medium. We hope that the piece of work presented here will contribute to further development of universal media without animal components.

## Declaration of Competing Interest

The authors declare that they have no known competing financial interests or personal relationships that could have appeared to influence the work reported in this paper.

## References

[1] Alm, J.J., Qian, Q., and Le Blanc, K. (2014) Clinical grade production of mesenchymal stromal cells. Editors: Clemens A. Van Blitterswijk, Jan De Boer, Tissue Engineering (Second Edition), Academic Press, ISBN 9780124201453.

[2] Antoniades H.N., Williams L.T. (1983). Human platelet-derived growth factor: structure and function. Fed. Proc..

[3] Baker M. (2016). Reproducibility: respect your cells. Nature.

[4] Belot N., Sim B., Longmore C., Roscoe L., Treasure C. (2017). Adaption of the keratinoSensTM skin sensitisation test to animal-product-free cell culture. ALTEX.

[5] Bieback K. (2013). Platelet lysate as replacement for fetal bovine serum in mesenchymal stromal cell cultures. Transf. Med. Hemother.

[6] Borgström B., Huang X., Hegardt C., Oredsson S., Strand D. (2017). Structure-activity relationships in salinomycin: cytotoxicity and phenotype selectivity of semi-synthetic derivatives. Chem. A Eur. J..

[7] Borgström B., Huang X., Pošta M., Hegardt C., Oredsson S., Strand D. (2013). Synthetic modification of salinomycin: selective o-acylation and biological evaluation. Chem. Com..

[8] Carrel A. (1912). On the permanent life of tissues outside of the organism. J. Exp. Med..

[9] Carrel A. (1913). Artificial activation of the growth in vitro of connective tissue. J. Exp. Med..

[10] Carrel A., Burrows M.T. (1910). Cultivation of adult tissues and organs outside of the body. J. Am. Med. Assoc..

[11] Carrel A., Burrows M.T. (1911). Cultivation of tissues in vitro and its technique. J. Exp. Med..

[12] Cassotta M., Bartnicka J.J., Pistollato F., Parvatam F., Weber T., D’Alessandro V., Ferreira Bastos L., Coecke S. (2022). A worldwide survey on the use of animal-derived materials and reagents in scientific experimentation. Eng. Life Sci..

[13] Crichton R.R., Wilmet S., Legssyer R., Ward R.J. (2002). Molecular and cellular mechanisms of iron homeostasis and toxicity in mammalian cells. J. Inorg. Biochem..

[14] Devireddy L.R., Myers M., Screven R., Liu Z., Boxer L. (2019). A serum-free medium formulation efficiently supports isolation and propagation of canine adipose-derived mesenchymal stem/stromal cells. PLoS ONE.

[15] Edmondson R., Broglie J.J., Adcock A.F., Yang L. (2014). Three-dimensional cell culture systems and their applications in drug discovery and cell-based biosensors. Assay. Drug Dev. Technol..

[16] Erdogan B., Ao M., White L.M., Means A.L., Brewer B.M., Yang L., Washington M.K., Shi C., Franco O.E., Weaver A.M., Hayward S.W., Li D., Webb D.J. (2017). Cancer-associated fibroblasts promote directional cancer cell migration by aligning fibronectin. J. Cell Biol..

[17] Francis G.L. (2010). Albumin and mammalian cell culture: implications for biotechnology applications. Cytotech.

[18] Friedl P., Gilmour D. (2009). Collective cell migration in morphogenesis regeneration and cancer. Nat. Rev. Mol. Cell Biol..

[19] Friedl P., Sahai E., Weiss S., Yamada K.M. (2012). New dimensions in cell migration. Nat. Rev. Mol. Cell Biol..

[20] Goos M., Lange P., Hanisch U.K., Prinz M., Scheffel J., Bergmann R., Ebert S., Nau R. (2007). Fibronectin is elevated in the cerebrospinal fluid of patients suffering from bacterial meningitis and enhances inflammation caused by bacterial products in primary mouse microglial cell cultures. J. Neurochem..

[21] Gstraunthaler G., Lindl T., van der Valk J. (2013). A plea to reduce or replace fetal bovine serum in cell culture media. Cytotechnol.

[22] Guetta-Terrier C., Monzo P., Zhu J., Long H., Venkatraman L., Zhou Y., Wang P., Chew S.Y., Mogilner A., Ladoux B., Gauthier N.C. (2015). Protrusive waves guide 3D cell migration along nanofibers. J. Cell Biol..

[23] Gupta P.B., Onder T.T., Jiang G., Tao K., Kuperwasser C., Weinberg R., Lander E.S. (2009). Identification of selective inhibitors of cancer stem cells by high-throughput screening. Cell.

[24] Hirsch C., Schildknecht S. (2019). In vitro research reproducibility: keeping up high standards. Front. Pharmacol..

[25] Huang X., Borgström B., Månsson L., Persson L., Oredsson S., Hegardt C., Strand D. (2014). Semisynthesis of SY-1 for investigation of breast cancer stem cell selectivity of C-ring-modified salinomycin analogues. ACS Chem. Biol..

[26] Huang X., Borgström B., Kempengren S., Persson L., Hegardt C., Strand D., Oredsson S. (2016). Breast cancer stem cell selectivity of synthetic nanomolar-active salinomycin analogs. BMC Cancer.

[27] Huang X., Borgström B., Stegmayr J., Abassi Y., Kruszyk M., Leffler H., Persson L., Albinsson S., Massoumi R., Scheblykon I.G., Hegardt C., Oredsson S., Strand D. (2018). The molecular basis for inhibition of stemlike cancer cells by salinomycin. ACS Cent. Sie..

[28] Hughes J.P., Rees S., Kalindjian S.B., Philpott K.L. (2011). Principles of early drug discovery. Br. J. Pharmacol..

[29] Huttala O., Vuorenpää H., Toimela T., Uotila J., Kuokkanen H., Ylikomi T., Sarkanen J.-R., Heinonen T. (2015). Human vascular model with defined stimulation medium - a characterization study. ALTEX.

[30] Imamura Y., Mukohara T., Shimono Y., Funakoshi Y., Chayahara N., Toyoda M., Kiyota N., Takao S., Kono S., Nakatsura T., Minami H. (2015). Comparison of 2D- and 3D-culture models as drug-testing platforms in breast cancer. Oncol. Rep..

[31] Ince T.A., Richardson A.L., Bell G.W., Saitoh M., Godar S., Karnoub A.E., Weinberg R.A. (2007). Transformation of different human breast epithelial cell types leads to distinct tumor phenotypes. Cancer Cell.

[32] Jensen C., Teng Y. (2020). Is it time to start transitioning from 2D to 3D cell culture. Front. Mol. Biosci..

[33] Jochems C.E., Van der Valk J.B., Stafleu F.R., Baumans V. (2002). The use of fetal bovine serum: ethical or scientific problem. Alt. Lab. Anim..

[34] Johnson J., Nowicki M.O., Lee C.H., Chiocca E.A., Viapiano M.S., Lawler S.E., Lannutti J.J. (2009). Quantitative analysis of complex glioma cell migration on electrospun polycaprolactone using time-lapse microscopy. Tissue Engin. Part C Methods.

[35] Joshi R.S., Kanugula S.S., Sudhir S., Pereira M.P., Jain S., Aghi M.K. (2021). The role of cancer-associated fibroblasts in tumor progression. Cancers.

[36] Kamlund S., Janicke B., Alm K., Oredsson S. (2020). Salinomycin treatment specifically inhibits cell proliferation of cancer stem cells revealed by longitudinal single cell tracking in combination with fluorescence microscopy. Appli. Sci..

[37] Kamlund S., Strand D., Janicke B., Alm K., Oredsson S. (2017). Influence of salinomycin treatment on division and movement of individual cancer cells cultured in normoxia or hypoxia evaluated with time-lapse digital holographic microscopy. Cell Cycle.

[38] Keiser H., LeRoy E.C., Udenfriend S., Sjoerdsma A. (1963). Collagen-like protein in human plasma. Science.

[39] Klein S.G., Alsolami S.M., Steckbauer A., Arossa S., Parry A.J., Ramos Mandujano G., Alsayegh K., Izpisua Belmonte J.C., Li M., Duarte C.M. (2021). A prevalent neglect of environmental control in mammalian cell culture calls for best practices. Nat. Biomed. Engin..

[40] Kojima Y., Acar A., Eaton E.N., Mellody K.T., Scheel C., Ben-Porath I., Onder T.T., Wang Z.C., Richardson A.L., Weinberg R.A., Orimo A. (2010). Autocrine TGF-β and stromal cell-derived factor-1 (SDF-1) signaling drives the evolution of tumor-promoting mammary stromal myofibroblasts. Proc. Nat. Acad. Sci..

[41] Magalhães Silva T., Andersson S., Sukumaran S.K., Marques M.P., Persson L., Oredsson S. (2013). Norspermidine and novel Pd(II) and Pt(II) polynuclear complexes of norspermidine as potential antineoplastic agents against breast cancer. PLoS ONE.

[42] Malakpour-Permlid A., Oredsson S. (2021). A novel 3D polycaprolactone high-throughput system for evaluation of toxicity in normoxia and hypoxia. Toxicol. Rep..

[43] Malakpour-Permlid A., Roci P., Fredlund E., Fält F., Önell E., Johansson F., Oredsson S. (2019). Unique animal friendly 3D culturing of human cancer and normal cells. Toxicol. Vitr..

[44] Nelson M.T., Short A., Cole S.L., Gross A.C., Winter J., Eubank T.D., Lannutti J.J. (2014). Preferential enhanced breast cancer cell migration on biomimetic electrospun nanofiber ‘cell highways’. BMC Cancer.

[45] Nirmalanandhan V.S., Duren A., Hendricks P., Vielhauer G., Sittampalam G.S. (2010). Activity of anticancer agents in a three-dimensional cell culture model. Assay. Drug Devt. Technol..

[46] OECD Guidance Document on Good In Vitro Method Practices (GIVIMP) (2018).

[47] Orimo A., Gupta P.B., Sgroi D.C., Arenzana-Seisdedos F., Delaunay T., Naeem R., Carey V.J., Richardson A.L., Weinberg R.A. (2005). Stromal fibroblasts present in invasive human breast carcinomas promote tumor growth and angiogenesis through elevated SDF-1/CXCL12 secretion. Cell.

[48] Ottosson M., Jakobsson A., Johansson F. (2017). Accelerated wound closure-differently organized nanofibers affect cell migration and hence the closure of artificial wounds in a cell based in vitro model. PLoS One.

[49] Pierzynowska K., Thomasson S., Oredsson S. (2022). Alpha-amylase inhibits cell proliferation and glucose uptake in human neuroblastoma cell lines. Biomed. Res. Int..

[50] Puck T.T., Cieciura S.J., Robinson A. (1958). Genetics of somatic mammalian cells. III. long-term cultivation of euploid cells from human and animal subjects. J. Ecp. Med.

[51] Ray A., Morford R.K., Provenzano P.P. (2018). Cancer stem cell migration in three‐dimensional aligned collagen matrices. Curr. Prot. Stem Cell Biol..

[52] Ristow H.J., Messmer T.O. (1988). Basic fibroblast growth factor and insulin‐like growth factor i are strong mitogens for cultured mouse keratinocytes. J. Cell. Physiol..

[53] Sakwe A.M., Koumangoye R., Goodwin S.J., Ochieng J. (2010). Fetuin-a (α2HS-glycoprotein) is a major serum adhesive protein that mediates growth signaling in breast tumor cells. J. Biol. Chem..

[54] Schedin P., Keely P.J. (2011). Mammary gland ECM remodeling stiffness and mechanosignaling in normal development and tumor progression. Cold Spring Harb. Perspect. Biol..

[55] Selby M., Delosh R., Laudeman J., Ogle C., Reinhart R., Silvers T., Lawrence S., Kinders R., Parchment R., Teicher B.A., Evans D.M. (2017). 3D models of the NCI60 cell lines for screening oncology compounds. SLAS DISCOVERY Adv. Life Sci. RD.

[56] Soria Sotillo W., Villagomez R., Smiljanic S., Huang X., Malakpour A., Kempengren S., Rodrigo G., Almanza G., Sterner O., Oredsson S. (2017). Anti-cancer stem cell activity of a sesquiterpene lactone isolated from *Ambrosia arborescens* and of a synthetic derivative. PLoS ONE.

[57] Souza A.G., Silva I.B.B., Campos-Fernandez E., Barcelos L.S., Souza J.B., Marangoni K., Goulart L.R., Alonso-Goulart V. (2018). Comparative assay of 2D and 3D cell culture models: proliferation gene expression and anticancer drug response. Curr. Pharm. Des..

[58] Stegmayr J., Zetterberg F., Carlsson M.C., Huang X., Shazrma G., Kahl-Knutson B., Schambye H., Nilsson U.J., Oredsson S., Leffler H. (2019). Extracellular and intracellular small-molecule galectin-3 inhibitors. Sci. Rep..

[59] Stiles C.D. (1983). The molecular biology of platelet-derived growth factor. Cell.

[60] Toimela T., Huttala O., Sabell E., Mannerström M., Sarkanen J.R., Ylikomi T., Heinonen T. (2017). Intra-laboratory validated human cell-based in vitro vasculogenesis/angiogenesis test with serum-free medium. Reprod. Toxicol..

[61] Toti A., Santi A., Pardella E., Nesi I., Tomasini R., Mello T., Paoli P., Caselli A., Cirri P. (2021). Activated fibroblasts enhance cancer cell migration by microvesicles-mediated transfer of Galectin-1. J. Cell Commun. Signal..

[62] van der Valk J., Brunner D., De Smet K., Fex Svenningsen Å., Honegger P., Knudsen L.E., Lindl T., Noraberg J., Price A., Scarino M.L., Gstraunthaler G. (2010). Optimization of chemically defined cell culture media - replacing fetal bovine serum in mammalian in vitro methods. Toxicol. Vitr..

[63] van der Valk J. (2022). Fetal bovine serum - a cell culture dilemma. Science.

[64] van der Valk J., Gstraunthaler G. (2017). Fetal bovine serum (FBS)-a pain in the dish? Alt. Lab. Anim..

[65] van der Valk J., Bieback K., Buta C., Cochrane B., Dirks W.G., Fu J., Hickman J.J., Hohensee C., Kolar R., Liebsch M., Pistollato F., Schulz M., Thieme D., Weber T., Wiest J., Winkler S., Gstraunthaler G. (2018). Fetal Bovine Serum (FBS). Past - Present - Future. ALTEX.

[66] Weber T., Wagner K. (2021). Replacing fetal bovine serum (FBS) in research and testing. ALTEX.

[67] Weber T., Wirths F., Brakebusch N., van der Valk J. (2021). Reply to comment animal welfare and ethics in the collection of fetal blood for the production of fetal bovine serum. ALTEX.

[68] Weber T., Wiest J., Oredsson S., Bieback K. (2022). Case studies exemplifying the transition to animal component-free cell culture. Alt. Lab. Anim..

[69] Wiegand C., Hipler U.C., Elsner P., Tittelbach J. (2021). Keratinocyte and fibroblast wound healing in vitro is repressed by non-optimal Cconditions but the reparative potential can be improved by water-filtered infrared A. Biomedicines.

[70] Wojtowicz A.M., Oliveira S., Carlson M.W., Zawadzka A., Rousseau C.F., Baksh D. (2014). The importance of both fibroblasts and keratinocytes in a bilayered living cellular construct used in wound healing. Wound Rep. Regen..

[71] Wong V.V., Ho K.W., Yap M.G. (2004). Evaluation of insulin-mimetic trace metals as insulin replacements in mammalian cell cultures. Cytotechnology.

[72] Yao T., Asayama Y. (2017). Animal‐cell culture media: history characteristics and current issues. Reproduc. Med. Biol..

