# Supplementary material for: A new animal product free defined medium for 2D and 3D culturing of normal and cancer cells to study cell proliferation and migration as well as dose response to chemical treatment
Source: Toxicol Rep. 2023 Apr 12;10:509–20. doi: 10.1016/j.toxrep.2023.04.001 (PMC10313884; doi:10.1016/j.toxrep.2023.04.001)
Supplement: Table S1 — Supplementary material [file mmc1.docx]

**Supplemental Figures**


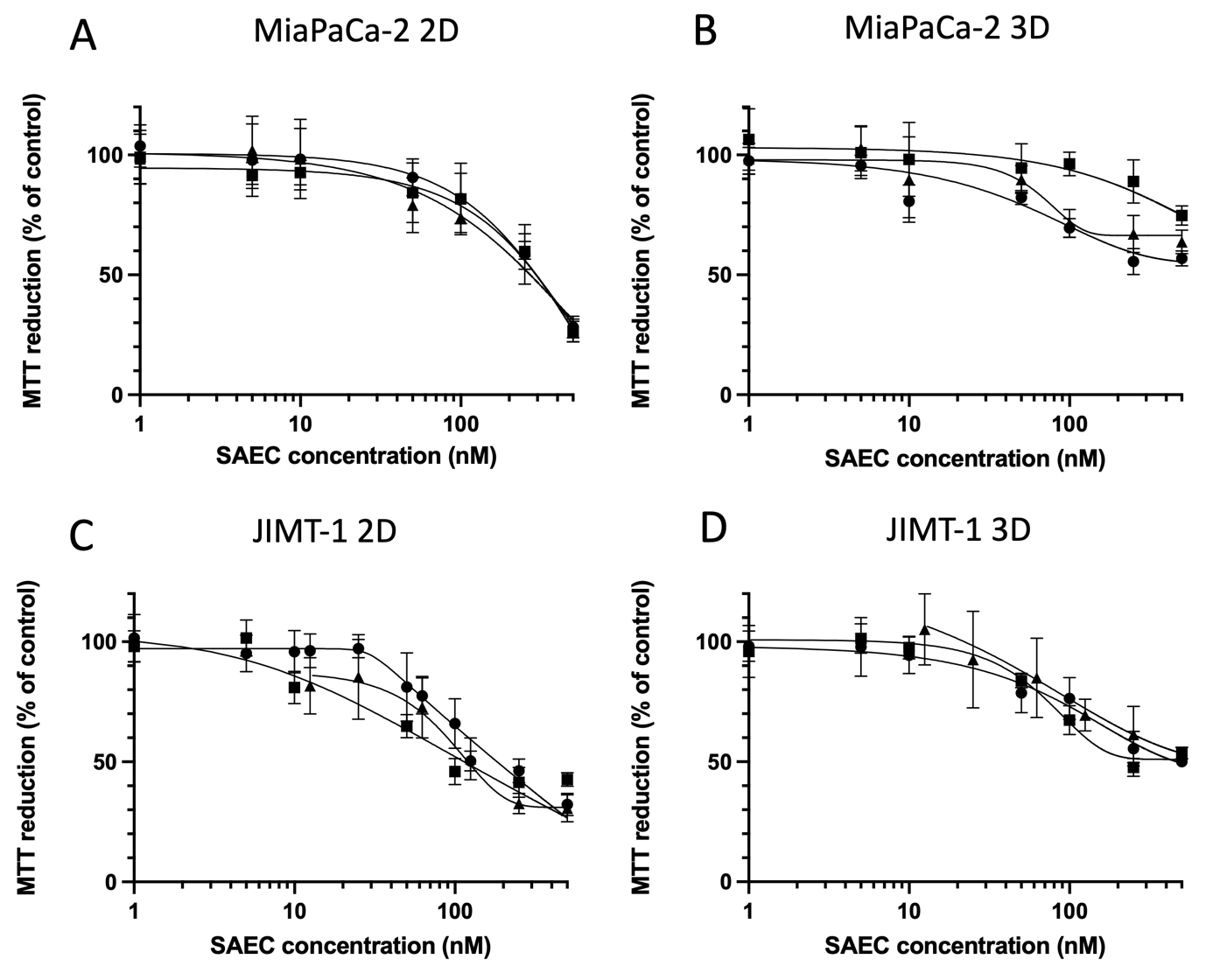


Supplemental Figure S1. Dose response curves for MiaPaCa-2 pancrea lapsc cancer cell (A and B) and JIMT-1 human breast cancer cells (C and D) in defined medium in 2D (A and C) and 3D (B and D) cultures treated with SAEC. The cells were seeded and incubated for 24 hour to allow attachment before addition of compound at the indicated concentrations. After 72 hours of incubation, the toxicity was evaluated using an MTT assay. Each data point is the mean ± SD of 6 wells. The curves are drawn in GraphPad Prism 9.


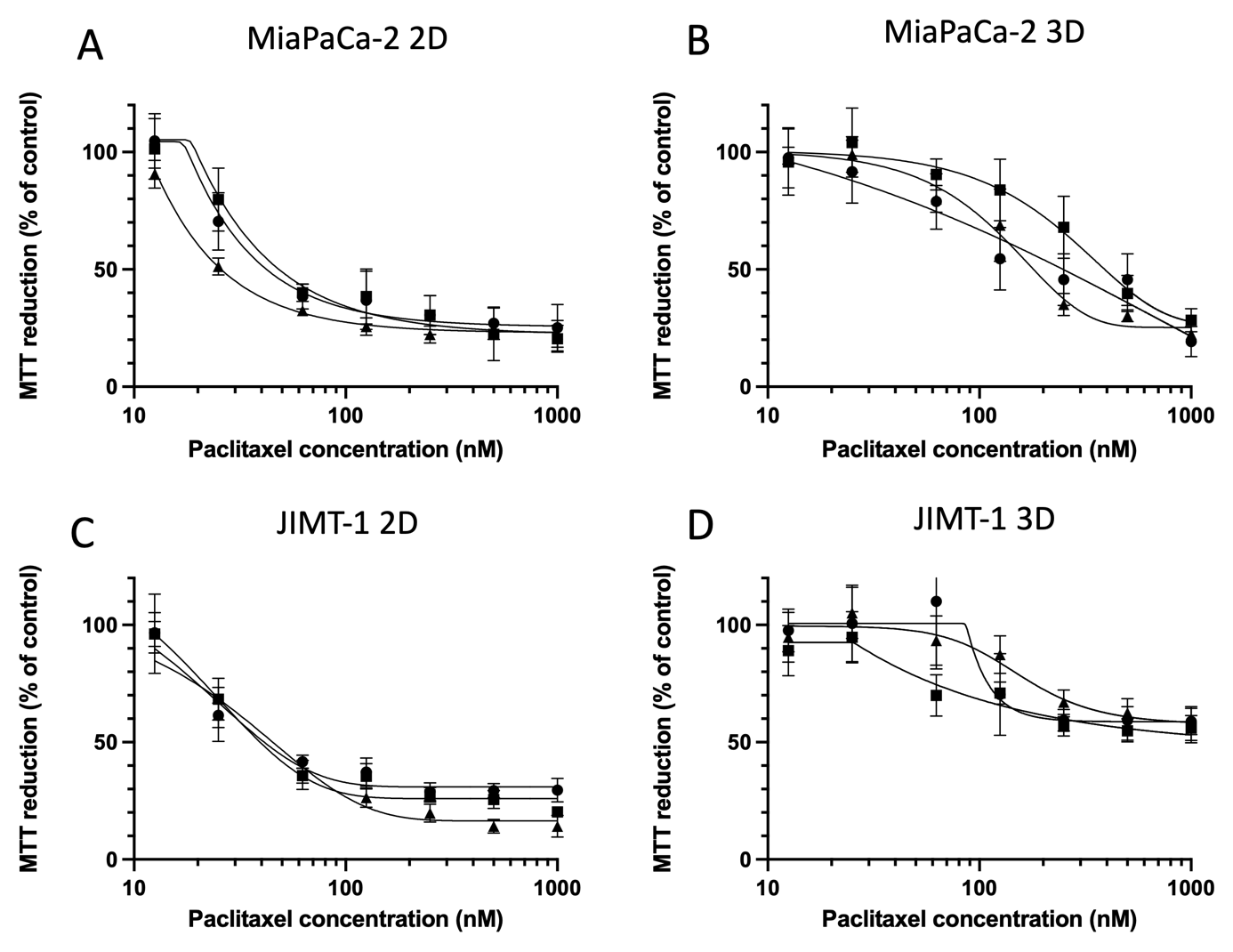


Supplemental Figure S2. Dose response curves for MiaPaCa-2 pancreatic cancer cell (A and B) and JIMT-1 human breast cancer cells (C and D) in defined medium in 2D (A and C) and 3D (B and D) cultures treated with paclitaxel. The cells were seeded and incubated for 24 hour to allow attachment before addition of compound at the indicated concentrations. After 72 hours of incubation, the toxicity was evaluated using an MTT assay. Each data point is the mean ± SD of 6 wells. The curves are drawn in GraphPad Prism 9.


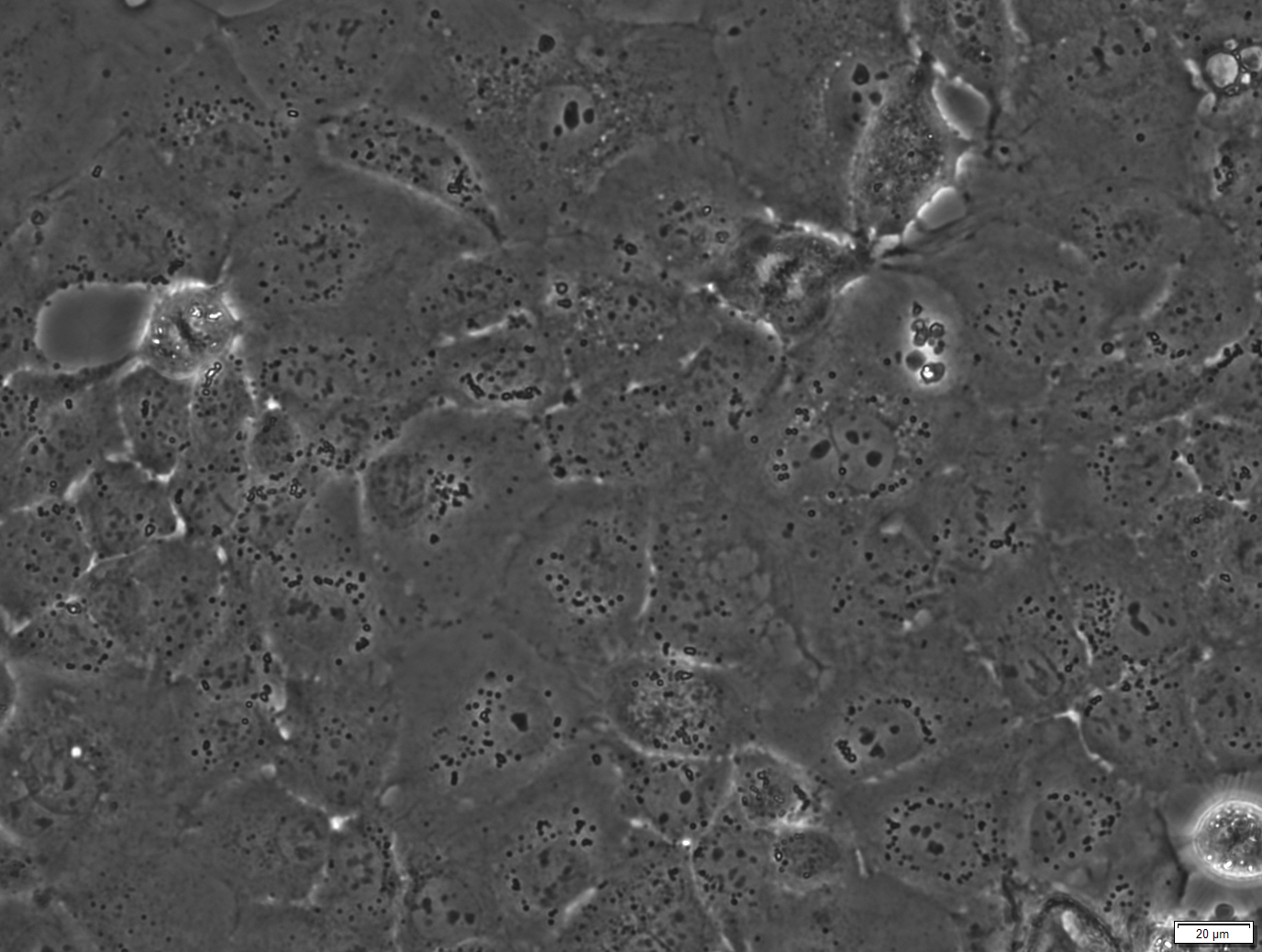


Supplemental Figure S3. Phase contrast microscopy image of CaCo-2 cells displaying the large cytoplasm of the cells and their tight growth.

**Supplemental Videos**

Supplemental Video S1. Phase contrast microscopy time-lapse video of keratinocytes cultured in the defined medium. Images were captured every 10 min for 72 hours.

Supplemental Video S2. Phase holographic microscopy time-lapse video of human colon cancer CaCo-2 cells cultured in the defined medium. Images were captured every 5 min for 72 hours.

Supplemental Video S3. Phase holographic microscopy time-lapse video of human breast cancer MDA-MB-231 cells cultured in the defined medium. Images were captured every 5 min for 72 hours.

Supplemental Video S4. Phase holographic microscopy time-lapse video of human breast cancer JIMT-1 cells cultured in the defined medium. Images were captured every 5 min for 72 hours.

Supplemental Video S5. Phase holographic microscopy time-lapse video of human breast cancer JIMT-1 spheroids cultured in the defined medium. Images were captured every 5 min for 72 hours. The capturing was initiated at the edge of the spheroid to capture cell migration into the viewing field.

Supplemental Video S6. Phase holographic microscopy time-lapse video of human pancreatic cancer MiaPaCa-2 spheroids cultured in the defined medium. Images were captured every 5 min for 72 hours. The capturing was initiated at the edge of the spheroid to capture cell migration into the viewing field.

Supplemental Video S7. Phase holographic microscopy time-lapse video of human breast cancer JIMT-1/CAF spheroids cultured in the defined medium. Images were captured every 5 min for 72 hours. The capturing was initiated at the edge of the spheroid to capture cell migration into the viewing field.

Supplemental Video S8. Phase holographic microscopy time-lapse video of human pancreatic cancer MiaPaCa-2/CAF spheroids cultured in the defined medium. Images were captured every 5 min for 48 hours. The capturing was initiated at the edge of the spheroid to capture cell migration into the viewing field.

Supplemental Video S9. Phase holographic microscopy time-lapse video of paclitaxel-treated (100 nM) human breast cancer JIMT-1 spheroids cultured in the defined medium. Images were captured every 5 min for 72 hours. The capturing was initiated at the edge of the spheroid to capture cell migration into the viewing field.

Supplemental Video S10. Phase holographic microscopy time-lapse video of paclitaxel-treated (100 nM) human breast cancer JIMT-1/CAF spheroids cultured in the defined medium. Images were captured every 5 min for 72 hours. The capturing was initiated at the edge of the spheroid to capture cell migration into the viewing field.

Supplemental Video S11. Phase contrast microscopy time-lapse video of human breast cancer JIMT-1 spheroids seeded on parallel PCL fibres in the defined medium. Images were captured every 10 min for 72 hours.

Supplemental Video S12. Phase contrast microscopy time-lapse video of human pancreatic cancer MiaPaCa-2 spheroids seeded on parallel PCL fibres in the defined medium. Images were captured every 10 min for 72 hours.

Supplementary Table S1. Sources and catalogue numbers of the components of the defined medium.

| **Medium component** | **Source** | **Product number** |
| --- | --- | --- |
| Basal medium: DMEM / Ham´s F12 | Biowest | L0090 |
| Optional extra buffer: HEPES | Sigma-Aldrich Sweden AB | 83264 |
| **Non-proteins** |  |  |
| All-trans retinoic acid | Sigma-Aldrich Sweden AB | R2625 |
| Alpha-tocopherol phosphate | Sigma-Aldrich Sweden AB | T2020 |
| para-Aminobenzoic acid | Sigma-Aldrich Sweden AB | A9878 |
| Ascorbic acid | Sigma-Aldrich Sweden AB | A4403 |
| Cholesterol | Sigma-Aldrich Sweden AB | C3045 |
| Choline chloride | Sigma-Aldrich Sweden AB | C7527 |
| Ergocalciferol | Sigma-Aldrich Sweden AB | E5750 |
| 17-beta Estradiol | Sigma-Aldrich Sweden AB | E2758 |
| Folic acid | Sigma-Aldrich Sweden AB | F8758 |
| Glutamine | Merck Biochrom | K0282 |
| Glutathione | Sigma-Aldrich Sweden AB | G6013 |
| Hydrocortisone | Sigma-Aldrich Sweden AB | H0888 |
| Hypoxanthine Na | Sigma-Aldrich Sweden AB | R2625 |
| I-inositol | Sigma-Aldrich Sweden AB | I7508 |
| Linoleic acid | Sigma-Aldrich Sweden AB | L1012 |
| Lipoic acid | Sigma-Aldrich Sweden AB | 07039 |
| Non-essential amino acids | Biowest | X0557 |
| *0*-Phosphoryl ethanolamine | Sigma-Aldrich Sweden AB | P0503 |
| Pyruvate Na | Merck Biochrom | L0473 |
| Ribose | Sigma-Aldrich Sweden AB | R9629 |
| Selenous acid | Sigma-Aldrich Sweden AB | 211176 |
| Thiamine HCl | Sigma-Aldrich Sweden AB | T1270 |
| Triiodothyronine | Sigma-Aldrich Sweden AB | T6397 |
| Uracil | Sigma-Aldrich Sweden AB | U1128 |
| Vitamin B12 | Sigma-Aldrich Sweden AB | V6629 |
| Xanthine | Sigma-Aldrich Sweden AB | X3627 |
| **Proteins (human)** |  |  |
| Basic fibroblast growth factor | Sigma-Aldrich Sweden AB | F3685 |
| Collagen | Sigma-Aldrich Sweden AB | C5533 |
| Epidermal growth factor | Sigma-Aldrich Sweden AB | E9644 |
| Fetuin A, α2-hs-glycoprotein | Sigma-Aldrich Sweden AB | G0516 |
| Fibronectin | EMD Millipor Corp. | FC010 |
| Insulin | Sigma-Aldrich Sweden AB | I9278 |
| Insulin-like growth factor 1 | ThermoFisher Scientific | PHG0078 |
| Laminin | Sigma-Aldrich Sweden AB | L6274 |
| Platelet-derived growth factor AA | PeproTech | 100-13A |
| Transferrin | Sigma-Aldrich Sweden AB | T3705 |
| Vitronectin | Stemcell Technologies | 7180 |
| Human serum albumin | Biowest | P6140 |
